# Poly[tris­(μ_4_-5-amino­isophthalato)diaqua­dilanthanum(III)]

**DOI:** 10.1107/S1600536808018849

**Published:** 2008-06-28

**Authors:** Tham Weng-Thim, Rohana Adnan, Hoong-Kun Fun, Samuel Robinson Jebas

**Affiliations:** a School of Chemical Science, Universiti Sains Malaysia, 11800 USM, Penang, Malaysia; b X-ray Crystallography Unit, School of Physics, Universiti Sains Malaysia, 11800 USM, Penang, Malaysia

## Abstract

The title compound, [La_2_(C_8_H_5_NO_4_)_3_(H_2_O)_2_]_*n*_, is a three-dimensional network coordination polymer in which each La^III^ ion is nine-coordinated by eight carboxyl­ate O atoms from six 5-amino­isophthalate ligands and one O atom from a water mol­ecule. One organic ligand lies on a twofold  rotation axis. O—H⋯O, O—H⋯N and N—H⋯O hydrogen bonds are observed in the crystal structure.

## Related literature

For related literature on metal carboxyl­ate complexes, see: Eddaoudi *et al.* (2002 ▶); Tao *et al.* (2000 ▶); Zheng *et al.* (2004 ▶). For related literature on aromatic polycarboxylic acids, see: Eddaoudi *et al.* (2000 ▶); Li *et al.* (1999 ▶); Lo *et al.* (2000 ▶); Qu *et al.* (2005 ▶); Rosi *et al.* (2002 ▶). For the coordination modes of lanthanides, see: Bond *et al.* (2000 ▶); Saleh *et al.* (1998 ▶). For bond length and angle data, see: Glunnlaugson *et al.* (2004 ▶); Zheng *et al.* (2003 ▶); Drew *et al.* (2000 ▶).
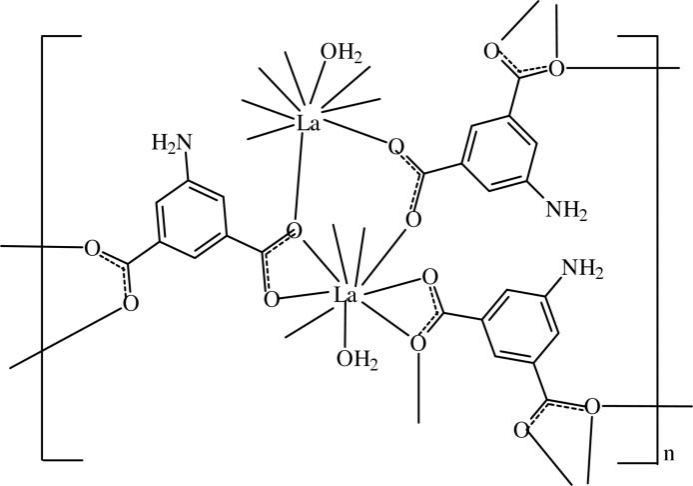


## Experimental

### 

#### Crystal data

[La_2_(C_8_H_5_NO_4_)_3_(H_2_O)_2_]
*M* *_r_* = 851.24
Orthorhombic, 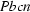

*a* = 12.2525 (3) Å
*b* = 8.0521 (2) Å
*c* = 25.6820 (6) Å
*V* = 2533.74 (11) Å^3^
*Z* = 4
Mo *K*α radiation
μ = 3.41 mm^−1^
*T* = 100.0 (1) K
0.36 × 0.31 × 0.06 mm

#### Data collection

Bruker SMART APEXII CCD area-detector diffractometer
Absorption correction: multi-scan (*SADABS*; Bruker, 2005 ▶) *T*_min_ = 0.313, *T*_max_ = 0.822
68438 measured reflections
7718 independent reflections
6892 reflections with *I* > 2σ(*I*)
*R*_int_ = 0.040

#### Refinement

*R*[*F* ^2^ > 2σ(*F* ^2^)] = 0.025
*wR*(*F* ^2^) = 0.074
*S* = 1.16
7718 reflections
208 parameters
5 restraints
H atoms treated by a mixture of independent and constrained refinement
Δρ_max_ = 1.54 e Å^−3^
Δρ_min_ = −1.24 e Å^−3^

### 

Data collection: *APEX2* (Bruker, 2005 ▶); cell refinement: *APEX2*; data reduction: *SAINT* (Bruker, 2005 ▶); program(s) used to solve structure: *SHELXTL* (Sheldrick, 2008 ▶); program(s) used to refine structure: *SHELXTL*; molecular graphics: *SHELXTL*; software used to prepare material for publication: *SHELXTL* and *PLATON* (Spek, 2003 ▶).

## Supplementary Material

Crystal structure: contains datablocks global, I. DOI: 10.1107/S1600536808018849/ci2610sup1.cif

Structure factors: contains datablocks I. DOI: 10.1107/S1600536808018849/ci2610Isup2.hkl

Additional supplementary materials:  crystallographic information; 3D view; checkCIF report

## Figures and Tables

**Table 1:** Hydrogen-bond geometry (Å, °)

*D*—H⋯*A*	*D*—H	H⋯*A*	*D*⋯*A*	*D*—H⋯*A*
O1*W*—H1*OW*⋯O5^i^	0.84	2.02	2.8354 (16)	164
O1*W*—H2*OW*⋯N1^ii^	0.84	2.02	2.817 (2)	157
N1—H1*N*1⋯O4^iii^	0.90 (1)	2.28 (2)	3.0929 (17)	150 (3)
N2—H1*N*2⋯O4^iv^	0.84 (1)	2.60 (2)	3.1953 (12)	129 (2)

Symmetry codes: (i) 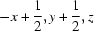; (ii) 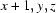; (iii) 
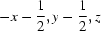
; (iv) 
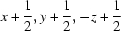
.

**Fig. 1.**
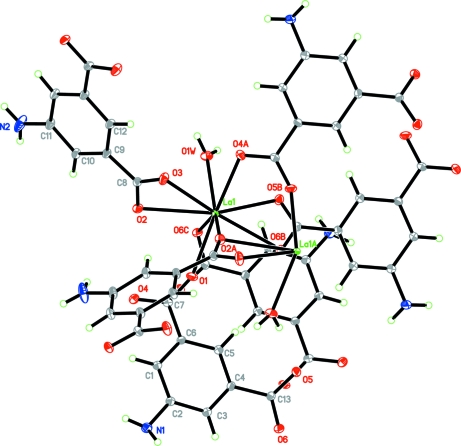
Part of the three-dimensional coordination polymer, showing the coordination environment of the LaIII ion. Displacement ellipsoids are drawn at the 50% probability level [symmetry codes: (A) 1/2-x, -1/2+y, z; (B) 1/2+x, 1/2-y, -z; (C) -x, 1-y, -z.]

**Fig. 2.**
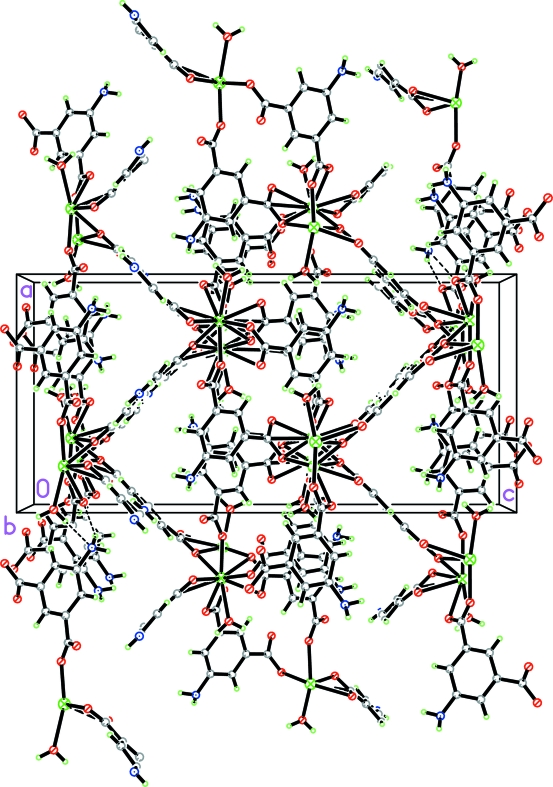
A view of part of the coordination polymer of the title compound. Hydrogen bonds are shown as dashed lines.

## supplementary crystallographic information

### Comment

In recent years, interest has been focused on metal carboxylate complexes due to their potential applications as material for molecular recognition, ion exchange, catalysis and luminescence (Eddaoudi *et al.*, 2002; Tao *et al.*, 2000; Zheng *et al.*, 2004). Aromatic polycarboxylic acids such as 1,4-benzenedicarboxylic acid and 1,3-benzenedicarboxylic acid are used extensively in the synthesis of this type of coordination polymer (Eddaoudi *et al.*, 2000; Li *et al.*, 1999; Lo *et al.*, 2000; Qu *et al.*, 2005; Rosi *et al.*, 2002).

Aminoisophthalic acid is a potential multidentate ligand with trifunctional group that may generate structures of higher dimensions containing networks or channels. It's two carboxylic groups may be completely or partially deprotonated and thus results in versatile coordination modes. Meanwhile, lanthanide ions are known for their ability to display diverse coordination modes and high coordination number (Bond *et al.*, 2000; Saleh *et al.*, 1998). In addition to that, hard acid lanthanide ions prefer to coordinate with carboxyl groups belonging to hard base. In this paper, we report the crystal structure of a polymeric coordination complex formed from hydrothermal reaction between trivalent lanthanum ion and aminoisophthalic acid.

In the crystal structure of the title compound, each La^III^ ion is nine-coordinated by eight carboxylate O atoms from six 5-aminoisophthalate ligands and one O atom from a water molecule. The La—O bond lengths [2.4076 (11) Å-2.8015 (12) Å] and bond angles around the La^III^ ion agree well with the values reported for related lanthanum complexes (Glunnlaugson *et al.*, 2004; Zheng *et al.*, 2003; Drew *et al.*, 2000).

The O—H···O, O—H···N and N—H···O hydrogen bonds are observed in the crystal structure (Fig. 2).

### Experimental

A mixture of 5-aminoisophthalic acid (0.273 g, 1.5 mmol), sodium hydroxide (0.120 g, 3 mmol) and distilled water (20 ml) was heated till boiling. The solution was left to cool and then poured into a 40 ml Teflon tube with La(NO_3_).6H_2_O (0.433 g, 1.0 mmol). The Teflon tube was sealed and heated at 403 K for 60 h, and then allowed to cool to room temperature. Pale clear-pink crystals obtained were filtered, washed with distilled water and left to dry in air (yield: 52% based on La).

### Refinement

H atoms of the water molecule were located initially in a difference Fourier map and then constrained to ride on the parent O atom, with O-H = 0.84 Å and *U*_iso_(H) = 0.018 Å^2^. The amino H atoms were located in a difference map and were refined with a N-H distance restraint of 0.85 (1) or 0.90 (1) Å. The remaining H atoms were positioned geometrically [C-H = 0.93 Å] and refined using a riding model with *U*_iso_(H) = 1.2U_eq_(C). The highest residual density peak is located 0.61 Å from La1 and the deepest hole is located 0.57 Å from La1.

### Crystal data

**Table:**

[La_2_(C_8_H_5_NO_4_)_3_(H_2_O)_2_]	*F*_000_ = 1640
*M**_r_* = 851.24	*D*_x_ = 2.221 Mg m^−^^3^
Orthorhombic, *P**b**c**n*	Mo *K*α radiation λ = 0.71073 Å
Hall symbol: -P 2n 2ab	Cell parameters from 9864 reflections
*a* = 12.2525 (3) Å	θ = 2.3–45.2º
*b* = 8.0521 (2) Å	µ = 3.41 mm^−^^1^
*c* = 25.6820 (6) Å	*T* = 100.0 (1) K
*V* = 2533.74 (11) Å^3^	Plate, pink
*Z* = 4	0.36 × 0.31 × 0.06 mm

### Data collection

**Table:**

Bruker SMART APEXII CCD area-detector diffractometer	7718 independent reflections
Radiation source: fine-focus sealed tube	6892 reflections with *I* > 2σ(*I*)
Monochromator: graphite	*R*_int_ = 0.040
*T* = 100.0(1) K	θ_max_ = 40.0º
φ and ω scans	θ_min_ = 1.6º
Absorption correction: multi-scan(SADABS; Bruker, 2005)	*h* = −22→21
*T*_min_ = 0.313, *T*_max_ = 0.822	*k* = −14→14
68438 measured reflections	*l* = −45→44

### Refinement

**Table:**

Refinement on *F*^2^	Secondary atom site location: difference Fourier map
Least-squares matrix: full	Hydrogen site location: inferred from neighbouring sites
*R*[*F*^2^ > 2σ(*F*^2^)] = 0.025	H atoms treated by a mixture of independent and constrained refinement
*wR*(*F*^2^) = 0.074	*w* = 1/[σ^2^(*F*_o_^2^) + (0.0368*P*)^2^ + 1.5159*P*] where *P* = (*F*_o_^2^ + 2*F*_c_^2^)/3
*S* = 1.16	(Δ/σ)_max_ = 0.001
7718 reflections	Δρ_max_ = 1.54 e Å^−^^3^
208 parameters	Δρ_min_ = −1.24 e Å^−^^3^
5 restraints	Extinction correction: none
Primary atom site location: structure-invariant direct methods	

### Special details

**Table:**

Experimental. The data was collected with the Oxford Cyrosystem Cobra low-temperature attachment.
Geometry. All e.s.d.'s (except the e.s.d. in the dihedral angle between two l.s. planes) are estimated using the full covariance matrix. The cell e.s.d.'s are taken into account individually in the estimation of e.s.d.'s in distances, angles and torsion angles; correlations between e.s.d.'s in cell parameters are only used when they are defined by crystal symmetry. An approximate (isotropic) treatment of cell e.s.d.'s is used for estimating e.s.d.'s involving l.s. planes.
Refinement. Refinement of *F*^2^ against ALL reflections. The weighted *R*-factor *wR* and goodness of fit *S* are based on *F*^2^, conventional *R*-factors *R* are based on *F*, with *F* set to zero for negative *F*^2^. The threshold expression of *F*^2^ > σ(*F*^2^) is used only for calculating *R*-factors(gt) *etc*. and is not relevant to the choice of reflections for refinement. *R*-factors based on *F*^2^ are statistically about twice as large as those based on *F*, and *R*- factors based on ALL data will be even larger.

### Fractional atomic coordinates and isotropic or equivalent isotropic displacement parameters (Å^2^)

**Table:**

	*x*	*y*	*z*	*U*_iso_*/*U*_eq_	
La1	0.305761 (5)	0.465168 (9)	0.094499 (3)	0.00725 (3)	
O1	0.11915 (9)	0.55822 (16)	0.09118 (5)	0.01427 (19)	
O2	0.29520 (8)	0.76545 (14)	0.14936 (5)	0.01260 (17)	
O3	0.36750 (11)	0.54193 (13)	0.18450 (5)	0.0161 (2)	
O4	0.03641 (8)	0.77482 (13)	0.12749 (5)	0.01283 (17)	
O5	−0.11694 (8)	0.16092 (13)	−0.01083 (4)	0.01261 (17)	
O6	−0.27525 (9)	0.27524 (13)	−0.03252 (4)	0.01193 (16)	
O1W	0.48255 (8)	0.60804 (14)	0.07751 (5)	0.01474 (18)	
H1OW	0.5115	0.6313	0.0488	0.018*	
H2OW	0.5309	0.6145	0.1009	0.018*	
N1	−0.35537 (11)	0.53130 (17)	0.15151 (6)	0.0164 (2)	
N2	0.5000	1.2332 (3)	0.2500	0.0265 (5)	
C1	−0.16182 (11)	0.57505 (17)	0.13169 (6)	0.0116 (2)	
H1	−0.1544	0.6421	0.1610	0.014*	
C2	−0.26282 (11)	0.50210 (18)	0.12013 (6)	0.0117 (2)	
C3	−0.27434 (10)	0.40880 (17)	0.07446 (6)	0.0112 (2)	
H3	−0.3426	0.3692	0.0647	0.013*	
C4	−0.18356 (10)	0.37549 (16)	0.04371 (6)	0.0102 (2)	
C5	−0.08197 (11)	0.44158 (16)	0.05646 (6)	0.0108 (2)	
H5	−0.0209	0.4155	0.0365	0.013*	
C6	−0.07239 (11)	0.54677 (16)	0.09915 (6)	0.0100 (2)	
C7	0.03462 (10)	0.63224 (16)	0.10726 (6)	0.01037 (19)	
C8	0.35819 (11)	0.69799 (17)	0.18250 (6)	0.0111 (2)	
C9	0.42765 (11)	0.80015 (16)	0.21813 (6)	0.0114 (2)	
C10	0.42555 (12)	0.97334 (16)	0.21897 (6)	0.0123 (2)	
H10	0.3747	1.0303	0.1989	0.015*	
C11	0.5000	1.0623 (3)	0.2500	0.0135 (3)	
C12	0.5000	0.7136 (2)	0.2500	0.0124 (3)	
H12	0.5000	0.5981	0.2500	0.015*	
C13	−0.19263 (10)	0.26435 (17)	−0.00240 (6)	0.0099 (2)	
H1N1	−0.399 (2)	0.443 (2)	0.1564 (11)	0.023 (7)*	
H1N2	0.5414 (19)	1.281 (3)	0.2714 (9)	0.028 (7)*	
H2N1	−0.342 (2)	0.571 (3)	0.1839 (6)	0.018 (6)*	

### Atomic displacement parameters (Å^2^)

**Table:**

	*U*^11^	*U*^22^	*U*^33^	*U*^12^	*U*^13^	*U*^23^
La1	0.00651 (4)	0.00736 (4)	0.00789 (4)	−0.00013 (2)	0.00015 (2)	−0.00035 (2)
O1	0.0076 (4)	0.0187 (5)	0.0165 (5)	0.0020 (3)	0.0003 (3)	−0.0007 (4)
O2	0.0128 (4)	0.0130 (4)	0.0120 (5)	0.0016 (3)	−0.0042 (3)	0.0008 (3)
O3	0.0223 (5)	0.0101 (4)	0.0160 (5)	0.0014 (3)	−0.0076 (4)	−0.0013 (3)
O4	0.0122 (4)	0.0111 (4)	0.0153 (5)	−0.0026 (3)	−0.0004 (3)	−0.0009 (3)
O5	0.0127 (4)	0.0129 (4)	0.0122 (5)	0.0030 (3)	−0.0022 (3)	−0.0024 (3)
O6	0.0107 (4)	0.0133 (4)	0.0118 (5)	0.0004 (3)	−0.0025 (3)	−0.0001 (3)
O1W	0.0108 (4)	0.0170 (5)	0.0163 (5)	−0.0029 (3)	0.0008 (3)	0.0023 (4)
N1	0.0108 (5)	0.0231 (6)	0.0153 (6)	−0.0028 (4)	0.0031 (4)	−0.0054 (4)
N2	0.0457 (13)	0.0095 (7)	0.0244 (11)	0.000	−0.0211 (10)	0.000
C1	0.0093 (5)	0.0122 (5)	0.0133 (6)	−0.0010 (3)	0.0002 (4)	−0.0023 (4)
C2	0.0098 (5)	0.0130 (5)	0.0123 (6)	−0.0006 (4)	0.0004 (4)	−0.0012 (4)
C3	0.0098 (4)	0.0119 (5)	0.0120 (6)	−0.0009 (3)	−0.0003 (4)	−0.0010 (4)
C4	0.0109 (5)	0.0092 (5)	0.0104 (6)	−0.0003 (3)	−0.0007 (4)	−0.0009 (4)
C5	0.0102 (4)	0.0107 (5)	0.0114 (6)	−0.0003 (3)	−0.0001 (4)	−0.0004 (4)
C6	0.0086 (5)	0.0095 (5)	0.0120 (6)	−0.0004 (3)	−0.0008 (4)	−0.0002 (4)
C7	0.0088 (4)	0.0114 (5)	0.0109 (5)	−0.0007 (3)	−0.0011 (4)	0.0010 (4)
C8	0.0119 (5)	0.0113 (5)	0.0101 (5)	0.0013 (3)	−0.0021 (4)	−0.0015 (4)
C9	0.0133 (5)	0.0108 (5)	0.0102 (5)	0.0003 (3)	−0.0032 (4)	−0.0008 (4)
C10	0.0164 (5)	0.0093 (5)	0.0113 (6)	0.0018 (3)	−0.0036 (4)	−0.0004 (4)
C11	0.0190 (8)	0.0098 (7)	0.0116 (8)	0.000	−0.0038 (6)	0.000
C12	0.0144 (7)	0.0098 (7)	0.0129 (8)	0.000	−0.0046 (6)	0.000
C13	0.0106 (5)	0.0100 (5)	0.0093 (5)	−0.0003 (3)	−0.0004 (4)	−0.0001 (4)

### Geometric parameters (Å, °)

**Table:**

La1—O1	2.4076 (11)	N1—H2N1	0.91 (1)
La1—O2^i^	2.4701 (11)	N2—C11	1.377 (3)
La1—O1W	2.4912 (10)	N2—H1N2	0.84 (1)
La1—O3	2.5093 (12)	C1—C6	1.397 (2)
La1—O5^ii^	2.5585 (11)	C1—C2	1.4016 (19)
La1—O4^i^	2.6089 (10)	C1—H1	0.93
La1—O6^iii^	2.6538 (11)	C2—C3	1.400 (2)
La1—O6^ii^	2.6955 (11)	C3—C4	1.3902 (19)
La1—O2	2.8015 (12)	C3—H3	0.93
O1—C7	1.2643 (18)	C4—C5	1.3927 (18)
O2—C8	1.2708 (17)	C4—C13	1.489 (2)
O2—La1^iv^	2.4701 (11)	C5—C6	1.390 (2)
O3—C8	1.2628 (17)	C5—H5	0.93
O4—C7	1.2604 (17)	C6—C7	1.4953 (18)
O4—La1^iv^	2.6089 (10)	C8—C9	1.4961 (19)
O5—C13	1.2651 (17)	C9—C12	1.3933 (17)
O5—La1^v^	2.5585 (11)	C9—C10	1.3949 (19)
O6—C13	1.2771 (17)	C10—C11	1.4071 (18)
O6—La1^iii^	2.6538 (11)	C10—H10	0.93
O6—La1^v^	2.6955 (11)	C11—C10^vi^	1.4071 (18)
O1W—H1OW	0.84	C12—C9^vi^	1.3933 (17)
O1W—H2OW	0.84	C12—H12	0.93
N1—C2	1.411 (2)	C13—La1^v^	3.0017 (14)
N1—H1N1	0.90 (1)		
			
O1—La1—O2^i^	75.36 (4)	C2—N1—H1N1	115.5 (19)
O1—La1—O1W	132.52 (4)	C2—N1—H2N1	116.3 (18)
O2^i^—La1—O1W	146.72 (4)	H1N1—N1—H2N1	105 (2)
O1—La1—O3	104.02 (4)	C11—N2—H1N2	117 (2)
O2^i^—La1—O3	77.64 (4)	C6—C1—C2	119.84 (13)
O1W—La1—O3	77.61 (4)	C6—C1—H1	120.1
O1—La1—O5^ii^	116.44 (4)	C2—C1—H1	120.1
O2^i^—La1—O5^ii^	113.96 (4)	C3—C2—C1	119.43 (12)
O1W—La1—O5^ii^	73.40 (4)	C3—C2—N1	119.13 (12)
O3—La1—O5^ii^	139.45 (4)	C1—C2—N1	121.25 (13)
O1—La1—O4^i^	153.93 (4)	C4—C3—C2	119.92 (12)
O2^i^—La1—O4^i^	78.67 (3)	C4—C3—H3	120.0
O1W—La1—O4^i^	71.56 (4)	C2—C3—H3	120.0
O3—La1—O4^i^	67.80 (4)	C3—C4—C5	120.51 (13)
O5^ii^—La1—O4^i^	76.42 (4)	C3—C4—C13	120.55 (11)
O1—La1—O6^iii^	66.41 (4)	C5—C4—C13	118.91 (12)
O2^i^—La1—O6^iii^	141.66 (3)	C6—C5—C4	119.58 (13)
O1W—La1—O6^iii^	69.74 (4)	C6—C5—H5	120.2
O3—La1—O6^iii^	113.64 (3)	C4—C5—H5	120.2
O5^ii^—La1—O6^iii^	82.01 (4)	C5—C6—C1	120.32 (12)
O4^i^—La1—O6^iii^	139.64 (3)	C5—C6—C7	117.66 (12)
O1—La1—O6^ii^	81.54 (4)	C1—C6—C7	121.98 (12)
O2^i^—La1—O6^ii^	71.63 (4)	O4—C7—O1	123.35 (13)
O1W—La1—O6^ii^	123.26 (4)	O4—C7—C6	119.47 (12)
O3—La1—O6^ii^	146.29 (3)	O1—C7—C6	117.12 (12)
O5^ii^—La1—O6^ii^	49.86 (3)	O3—C8—O2	120.49 (13)
O4^i^—La1—O6^ii^	92.45 (3)	O3—C8—C9	118.09 (12)
O6^iii^—La1—O6^ii^	99.18 (3)	O2—C8—C9	121.33 (12)
O1—La1—O2	72.86 (4)	O3—C8—La1	55.20 (8)
O2^i^—La1—O2	104.59 (4)	O2—C8—La1	68.49 (8)
O1W—La1—O2	74.31 (4)	C9—C8—La1	157.57 (9)
O3—La1—O2	48.55 (3)	C12—C9—C10	120.17 (13)
O5^ii^—La1—O2	141.45 (3)	C12—C9—C8	116.50 (12)
O4^i^—La1—O2	112.21 (3)	C10—C9—C8	123.28 (13)
O6^iii^—La1—O2	67.38 (3)	C9—C10—C11	120.37 (14)
O6^ii^—La1—O2	154.13 (3)	C9—C10—H10	119.8
C7—O1—La1	155.94 (10)	C11—C10—H10	119.8
C8—O2—La1^iv^	164.67 (10)	N2—C11—C10	120.59 (9)
C8—O2—La1	86.55 (8)	N2—C11—C10^vi^	120.59 (9)
La1^iv^—O2—La1	107.35 (4)	C10—C11—C10^vi^	118.81 (18)
C8—O3—La1	100.39 (9)	C9^vi^—C12—C9	119.99 (18)
C7—O4—La1^iv^	114.46 (9)	C9^vi^—C12—H12	120.0
C13—O5—La1^v^	97.67 (9)	C9—C12—H12	120.0
C13—O6—La1^iii^	121.94 (9)	O5—C13—O6	121.52 (13)
C13—O6—La1^v^	90.94 (8)	O5—C13—C4	118.50 (12)
La1^iii^—O6—La1^v^	105.27 (4)	O6—C13—C4	119.99 (12)
La1—O1W—H1OW	128.6	O5—C13—La1^v^	57.64 (7)
La1—O1W—H2OW	120.9	O6—C13—La1^v^	63.88 (8)
H1OW—O1W—H2OW	108.3	C4—C13—La1^v^	175.99 (9)
			
O2^i^—La1—O1—C7	81.4 (3)	La1—O3—C8—O2	21.93 (16)
O1W—La1—O1—C7	−77.4 (3)	La1—O3—C8—C9	−154.83 (11)
O3—La1—O1—C7	8.5 (3)	La1^iv^—O2—C8—O3	−174.7 (3)
O5^ii^—La1—O1—C7	−168.8 (2)	La1—O2—C8—O3	−19.25 (14)
O4^i^—La1—O1—C7	76.4 (3)	La1^iv^—O2—C8—C9	1.9 (5)
O6^iii^—La1—O1—C7	−101.5 (3)	La1—O2—C8—C9	157.41 (13)
O6^ii^—La1—O1—C7	154.5 (3)	La1^iv^—O2—C8—La1	−155.5 (4)
O2—La1—O1—C7	−29.2 (3)	O1—La1—C8—O3	120.56 (10)
C13^ii^—La1—O1—C7	172.0 (3)	O2^i^—La1—C8—O3	45.09 (10)
C8—La1—O1—C7	−13.0 (3)	O1W—La1—C8—O3	−104.47 (10)
O1—La1—O2—C8	138.62 (9)	O5^ii^—La1—C8—O3	−97.27 (11)
O2^i^—La1—O2—C8	69.33 (7)	O4^i^—La1—C8—O3	−33.35 (10)
O1W—La1—O2—C8	−76.18 (9)	O6^iii^—La1—C8—O3	−173.07 (10)
O3—La1—O2—C8	10.99 (8)	O6^ii^—La1—C8—O3	58.48 (19)
O5^ii^—La1—O2—C8	−110.23 (9)	O2—La1—C8—O3	159.76 (15)
O4^i^—La1—O2—C8	−14.18 (9)	C13^ii^—La1—C8—O3	−82.2 (2)
O6^iii^—La1—O2—C8	−150.35 (9)	O1—La1—C8—O2	−39.20 (9)
O6^ii^—La1—O2—C8	147.23 (9)	O2^i^—La1—C8—O2	−114.68 (7)
C13^ii^—La1—O2—C8	−148.28 (9)	O1W—La1—C8—O2	95.77 (9)
O1—La1—O2—La1^iv^	−47.98 (4)	O3—La1—C8—O2	−159.76 (15)
O2^i^—La1—O2—La1^iv^	−117.27 (6)	O5^ii^—La1—C8—O2	102.97 (9)
O1W—La1—O2—La1^iv^	97.22 (5)	O4^i^—La1—C8—O2	166.88 (8)
O3—La1—O2—La1^iv^	−175.61 (7)	O6^iii^—La1—C8—O2	27.17 (8)
O5^ii^—La1—O2—La1^iv^	63.17 (6)	O6^ii^—La1—C8—O2	−101.28 (16)
O4^i^—La1—O2—La1^iv^	159.22 (4)	C13^ii^—La1—C8—O2	118.03 (17)
O6^iii^—La1—O2—La1^iv^	23.04 (4)	O1—La1—C8—C9	−159.9 (3)
O6^ii^—La1—O2—La1^iv^	−39.37 (10)	O2^i^—La1—C8—C9	124.7 (3)
C13^ii^—La1—O2—La1^iv^	25.12 (10)	O1W—La1—C8—C9	−24.9 (2)
C8—La1—O2—La1^iv^	173.40 (11)	O3—La1—C8—C9	79.6 (3)
O1—La1—O3—C8	−62.50 (10)	O5^ii^—La1—C8—C9	−17.7 (3)
O2^i^—La1—O3—C8	−133.75 (10)	O4^i^—La1—C8—C9	46.2 (3)
O1W—La1—O3—C8	68.68 (10)	O6^iii^—La1—C8—C9	−93.5 (3)
O5^ii^—La1—O3—C8	113.71 (10)	O6^ii^—La1—C8—C9	138.1 (2)
O4^i^—La1—O3—C8	143.60 (11)	O2—La1—C8—C9	−120.7 (3)
O6^iii^—La1—O3—C8	7.57 (11)	C13^ii^—La1—C8—C9	−2.6 (4)
O6^ii^—La1—O3—C8	−158.30 (8)	O3—C8—C9—C12	1.7 (2)
O2—La1—O3—C8	−11.23 (8)	O2—C8—C9—C12	−175.02 (12)
C13^ii^—La1—O3—C8	152.93 (9)	La1—C8—C9—C12	−64.5 (3)
C6—C1—C2—C3	−3.4 (2)	O3—C8—C9—C10	179.15 (15)
C6—C1—C2—N1	−178.49 (14)	O2—C8—C9—C10	2.4 (2)
C1—C2—C3—C4	6.3 (2)	La1—C8—C9—C10	112.9 (2)
N1—C2—C3—C4	−178.49 (14)	C12—C9—C10—C11	3.2 (2)
C2—C3—C4—C5	−3.4 (2)	C8—C9—C10—C11	−174.17 (12)
C2—C3—C4—C13	174.65 (13)	C9—C10—C11—N2	178.42 (11)
C3—C4—C5—C6	−2.5 (2)	C9—C10—C11—C10^vi^	−1.58 (11)
C13—C4—C5—C6	179.43 (13)	C10—C9—C12—C9^vi^	−1.58 (11)
C4—C5—C6—C1	5.4 (2)	C8—C9—C12—C9^vi^	175.93 (14)
C4—C5—C6—C7	−172.14 (13)	La1^v^—O5—C13—O6	0.96 (15)
C2—C1—C6—C5	−2.4 (2)	La1^v^—O5—C13—C4	−178.75 (10)
C2—C1—C6—C7	174.99 (13)	La1^iii^—O6—C13—O5	−109.60 (13)
La1^iv^—O4—C7—O1	29.15 (18)	La1^v^—O6—C13—O5	−0.90 (14)
La1^iv^—O4—C7—C6	−147.91 (10)	La1^iii^—O6—C13—C4	70.10 (15)
La1—O1—C7—O4	42.8 (3)	La1^v^—O6—C13—C4	178.80 (11)
La1—O1—C7—C6	−140.0 (2)	La1^iii^—O6—C13—La1^v^	−108.70 (8)
C5—C6—C7—O4	147.88 (14)	C3—C4—C13—O5	−138.65 (14)
C1—C6—C7—O4	−29.6 (2)	C5—C4—C13—O5	39.5 (2)
C5—C6—C7—O1	−29.36 (19)	C3—C4—C13—O6	41.6 (2)
C1—C6—C7—O1	153.14 (14)	C5—C4—C13—O6	−140.26 (14)

Symmetry codes: (i) −*x*+1/2, *y*−1/2, *z*; (ii) *x*+1/2, −*y*+1/2, −*z*; (iii) −*x*, −*y*+1, −*z*; (iv) −*x*+1/2, *y*+1/2, *z*; (v) *x*−1/2, −*y*+1/2, −*z*; (vi) −*x*+1, *y*, −*z*+1/2.

### Hydrogen-bond geometry (Å, °)

**Table:**

*D*—H···*A*	*D*—H	H···*A*	*D*···*A*	*D*—H···*A*
O1W—H1OW···O5^iv^	0.84	2.02	2.8354 (16)	164
O1W—H2OW···N1^vii^	0.84	2.02	2.817 (2)	157
N1—H1N1···O4^viii^	0.90 (1)	2.28 (2)	3.0929 (17)	150 (3)
N2—H1N2···O4^ix^	0.84 (1)	2.60 (2)	3.1953 (12)	129 (2)

Symmetry codes: (iv) −*x*+1/2, *y*+1/2, *z*; (vii) *x*+1, *y*, *z*; (viii) −*x*−1/2, *y*−1/2, *z*; (ix) *x*+1/2, *y*+1/2, −*z*+1/2.
